# P-1744. Impact of a Multimodal Antimicrobial Stewardship Intervention on Fluoroquinolone Usage for Antimicrobial Prophylaxis before Urologic Procedures at South Texas Veterans Health Care System

**DOI:** 10.1093/ofid/ofae631.1907

**Published:** 2025-01-29

**Authors:** Gertrude Kinyua, Gaielle Harb, Teri L Hopkins, Christopher R Frei, Vidal M Mendoza, Jose Cadena-Zuluaga, Elizabeth Walter

**Affiliations:** South Texas Veterans Healthcare System, San Antonino, TX, US / UT Health San Antonio, San Antonio, TX, US /UT Austin College of Pharmacy, Austin, TX, US, San Antonio, Texas; o South Texas Veterans Healthcare System, San Antonino, TX, US/ UT Health San Antonio Long School of Medicine, San Antonio, TX, US/ UT Austin College of Pharmacy, Austin, TX, US, San Antonio, Texas; South Texas Veterans Health Care System, San Antonio, Texas; University of Texas, San Antonio, Texas; UT College of Pharmacy, San Antonio, Texas; South Texas Veterans Healthcare System, San Antonino, TX, US / UT Health San Antonio Long School of Medicine, San Antonio, TX, US, San Antonio, Texas; South Texas Veterans Health Care System, San Antonio, Texas

## Abstract

**Background:**

Fluoroquinolones (FQ) are frequently prescribed for urologic procedure prophylaxis, despite being associated with a myriad of adverse events and increasing rates of resistant bacteria. The purpose of this project was to evaluate the impact of a multimodal antimicrobial stewardship intervention on FQ usage for antimicrobial prophylaxis in urologic procedures in an outpatient setting.

**Figure:**
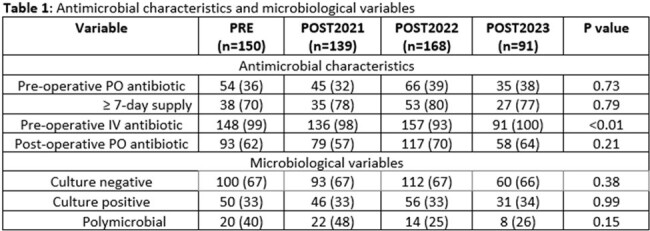

**Methods:**

This was a single-center, retrospective, cohort study of patients at the South Texas VA who underwent urologic procedures from December 1, 2020 to February 28, 2024. Initiatives conducted during this period included academic detailing, generation of provider specific FQ usage reports for review by the chief of urology, and prospective reviews of preoperative urine cultures followed by feedback on pathogen-directed therapy. The study was composed of a pre-intervention cohort and 3 post-intervention cohorts. The primary outcome was FQ days of therapy (DOT). Secondary outcomes included number of ≥3-day versus 1-2-day supply prescriptions, percentage of inappropriate prescriptions, rate of 30-day postoperative urinary tract infection (UTI), surgical site infection (SSI) and blood stream infection (BSI), emergence of FQ resistance within 1 year after procedure, and *Clostridioides difficile* infection (CDI) 30 days after completion of antimicrobial prophylaxis.

**Figure:**
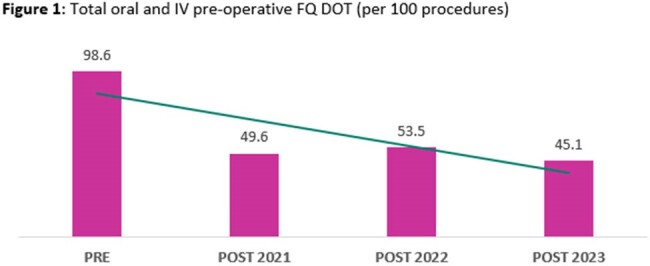

**Results:**

Data from 548 patients were included for analysis (150 PRE; 139 POST2021; 168 POST2022; 91 POST2023). The majority of patients were male (94.5%), with a median age of 67 (IQR, 59-74) years. Antimicrobial and microbiological characteristics are described in Table 1. A significant decrease in pre-operative FQ DOT/100 procedures was observed between the pre- and post-intervention groups (Fig 1). This decrease was driven by a reduction of pre-operative IV FQ usage (Fig 3) with minimal impact observed with pre-operative PO FQ usage (Fig 2). No difference was observed in percentage of inappropriate prescriptions which was entirely driven by inappropriate day supply. No significant differences were observed in the secondary outcomes assessed.

**Figure:**
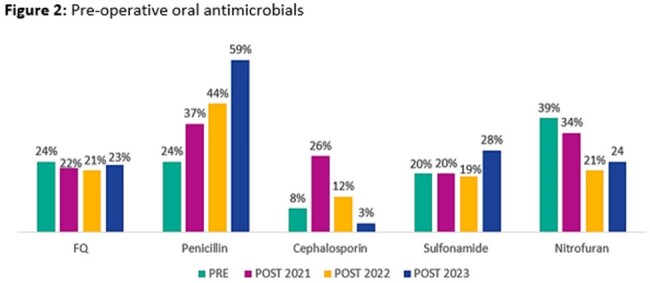

**Conclusion:**

A multimodal stewardship initiative decreased overall FQ usage prior to urologic procedures.

**Figure:**
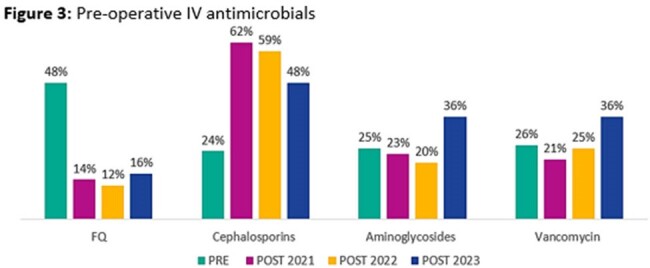

**Disclosures:**

**Christopher R. Frei, PharmD, FCCP, BCPS**, AstraZeneca: Grant/Research Support

